# Altered nutrition behavior during COVID-19 pandemic lockdown in young adults

**DOI:** 10.1007/s00394-020-02435-6

**Published:** 2020-12-01

**Authors:** Bruno C. Huber, Julius Steffen, Jenny Schlichtiger, Stefan Brunner

**Affiliations:** 1 Department of Medicine I, Ludwig-Maximilians-University Munich, University Hospital, Campus Innenstadt, Ziemssenstrasse 1, 80336 Munich, Germany; 2 Munich Heart Alliance (MHA), Partner Site Munich, DZHK (German Centre for Cardiovascular Research), Marchioninistrasse 15, 81377 Munich, Germany

**Keywords:** Students, COVID-19 pandemic lockdown, SARS-CoV-2, Cardiovascular prevention, Dietary habits, Eating behavior

## Abstract

**Purpose:**

The COVID-19 pandemic and the implemented lockdown strongly impact on everyone’s daily life. Stressful situations are known to alter eating habits and increase the risk for obesity. In our study, we aimed to investigate the effect of the lockdown measures on nutrition behavior among young adults.

**Methods:**

In this cross-sectional study, we enrolled 1964 voluntary participants from Bavarian universities. All participants were asked to complete an online questionnaire, semi-quantitatively evaluating the amount and type of food before and during pandemic lockdown. Study subjects were inquired to give information about acquisition and food procurement. The primary outcome was the change in food amount, secondary outcomes included alterations of food composition and procurement.

**Results:**

Our study cohort (mean age 23.3 ± 4.0 years, 28.5% male) had a mean body mass index of 22.1 ± 4.5 kg/m^2^. The overall food amount increased in 31.2% of participants (*n* = 610) during lockdown and decreased in 16.8% (*n* = 328). A multinominal regression model revealed that an increased food intake was less likely in male participants (OR, 0.7 [CI 0.6–0.9]) and more likely with increasing BMI (OR, 1.4 [CI 1.3–2.0]), increased sports activity (OR, 1.3 [CI 1.2–1.8]), augmented mental stress (OR 1.4 [1.1–1.7]), and an alteration of alcohol consumption (reduced alcohol amount, OR, 1.4 [CI 1.1–1.7], increased alcohol, OR, 1.9 [CI 1.4–2.5]). Increase in food intake was mainly triggered by consumption of bread (increased in 46.8%, *n* = 284) and confectionary (increased in 64.4%, *n* = 389).

**Conclusion:**

The COVID-19 pandemic lockdown significantly affected eating habits in young adults. Further investigation to evaluate long-term effects on weight change and comorbidities are warranted.

**Electronic supplementary material:**

The online version of this article (10.1007/s00394-020-02435-6) contains supplementary material, which is available to authorized users.

## Introduction

In December 2019, the new coronavirus SARS-CoV-2 (severe acute respiratory syndrome coronavirus 2) emerged in the Chinese city of Wuhan, causing an atypical pneumonia named COVID-19 [1]. From China, the new virus rapidly spread throughout the world and developed into a pandemic. The exponential growth of COVID-19 cases challenged the capacity of health-care systems in an unprecedented manner, so that governments were forced to impose a lockdown with restrictions concerning all areas of life.

**Fig. 1:**
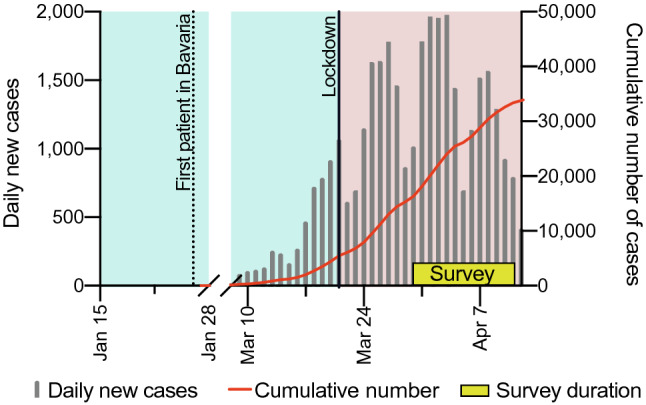
Daily new cases in Bavaria: timeline showing the number of new confirmed infections with SARS-CoV-2 in Bavaria per day (gray bars) and the cumulative number of cases (red line). The first confirmed case of COVID-19 in Bavaria was on the 27th of January (dotted line), which was also the first case in Germany. Lockdown was implemented by the local authorities in Bavaria on the 21st of March (line). Study participants were asked to compare their eating habits before the lockdown (pastel green area) to during the study period (yellow box) since implementation of the lockdown (pastel red area)

On March 21, the German federal state Bavaria imposed a countrywide lockdown involving closure of shops, restaurants, bars, markets and beer gardens (Fig. 1). Alongside the Saarland, it was the first region in Germany to introduce these measures. All German states introduced similar measures one after another, mainly enforcing social distancing by closure of restaurants, sports and cultural facilities as well as by prohibiting interaction of people from different households. Food acquisition was restricted to supermarkets, grocery stores and delivery services.

Eating behavior is strongly influenced by cultural and social aspects: sharing a meal with friends, family or colleagues is a common habit, which is deeply rooted in our culture and society [2]. It is well known that eating behavior is different when eating alone compared to eating with other people [3]. Furthermore, dietary choices converge with those of close social connections, which may be explained by shared social and cultural expectations and norms [4].

On the other hand, eating behavior has a major impact on health condition and the development of various diseases. Dietary habits are crucial for the development of cardiovascular diseases and even for all-cause mortality. Therefore, a so-called healthy diet plays a key role in both, primary and secondary cardiovascular prevention, and is given a class I recommendation in current guidelines [5]. Further, unhealthy diet patterns may have pro-inflammatory properties with the risk of the development and aggravation of inflammatory diseases including pulmonary infections [6].

So far, data on behavioral and dietary changes during the COVID-19 lockdown are scarce and are mainly limited to observational studies for certain patient cohorts [7] and regions [8]. However, the impact of lockdown measures on healthy young adults has not been addressed yet.

We hypothesized that lockdown measures would influence nutrition behavior among young adults. Therefore, we aimed to explore how food consumption changed during lockdown and to determine which factors impacted these changes.

## Methods

### Study oversight

The COVID-19 Pandemic Lockdown in Young Adults (COLA) trial was a cross-sectional study, performing an online survey among young adults. The ethics committee at the Ludwig-Maximilians-University (LMU) of Munich, Germany, approved the study. Participants of the online survey were informed and consented to all recorded data being used for study purposes. The study was registered at clinicaltrials.gov with the accession identifier NCT04361877.

Primary outcome was the self-reported change in food consumption since introduction of lockdown. Secondary outcomes were the change in food procurement and composition of food.

### Survey conduction

The online survey was organized and conducted as described before. The questionnaire was prepared using EvaSys (Electric Paper Evaluationssysteme GmbH, Lüneburg, Germany). The large-scale online survey consisted of 10 questions with 18 items (2 multiple choice items, 3 open ended, and 13 closed-ended items, see Appendix). The first part of the questionnaire collected information on participants’ demographics (gender, weight, height, age). The body mass index (BMI) was categorized based on the distribution of the data (< 20 kg/m^2^; 20–25 kg/m^2^; > 25 kg/m^2^). In the second part, participants answered questions about their lifestyle habits, including food consumption, sports activity, smoking and alcohol consumption since implementation of lockdown in Bavaria (21st of March 2020) compared to the time before on a 3-level scale (“decreased”, “unchanged”, “increased”). Similarly, they were asked to quantify the overall amount of food consumed as well as different types of food (i.e., bread, dairy, vegetables, fruit, meat, confectionary) they consumed compared to before lockdown. Additionally, changes in food procurement (convenient/delivery/restaurant, cafeteria, home-cooked) were recorded. An English translation of the questionnaire is attached in the Supplementary Appendix.

The questionnaire was distributed via accessible mailing lists of students at LMU Munich (*n* = 7752). Additionally, the main offices of five other Bavarian universities were contacted with the request to forward the survey to their students (students contacted: *n* = 500). The survey was active for 2 weeks in March and April 2020 (Fig. 1).

### Statistical analysis of survey data

SPSS version 25 for Windows (SPSS Inc., Chicago, USA), Prism 8 for mac version 8.4.2 (GraphPad Software, San Diego, USA) and Adobe Illustrator version 24.0.3 (Adobe Inc., San Jose, USA) were used for statistical analysis and design of graphs.

Analyses were performed stratified for participants who declared altered eating habits (eating “more” or “less”, respectively) since the lockdown. Participants that were over the age of 50 years (*n* = 16) were excluded from the analysis. There were no pre-specified subgroup analyses and the study was intended to be merely descriptive.

For data description, absolute and relative frequencies were calculated. Categorical variables (gender, age, BMI, Corona-test results) were compared using Chi-squared test. Non-categorial variables (age, BMI) were compared using Kruskal–Wallis test. Normal distribution was tested using the Kolmogorov–Smirnov test. Statistical significance was determined at p < 0.05. Using bivariate analyses, potential influence factors on the outcome variable (changes in food consumption since lockdown). Subsequently, all variables significantly associated with food consumption were included into a multinomial logistic regression model. To adjust for potential confounding, age was included into the regression model, though it did not show a significant correlation with the outcome in the bivariate analyses.

## Results

### Study demographics

A total of 1980 students from six different Bavarian universities participated in the large-scale online survey (response rate, 24%). Due to the age restriction, *n* = 16 (0.008%) were excluded from the analyses. Only participants with available data on change in the amount of consumed food were analyzed, which led to a study sample of *n* = 1957. Participating students had a mean age of 23.4 ± 4.0 years (mean ± standard deviation), 71.5% (*n* = 1,383) were female, and most participants had a normal body mass index (BMI) (22.1 ± 4.5 kg/m^2^).

### Changes in food procurement due to COVID-19 lockdown

**Fig. 2:**
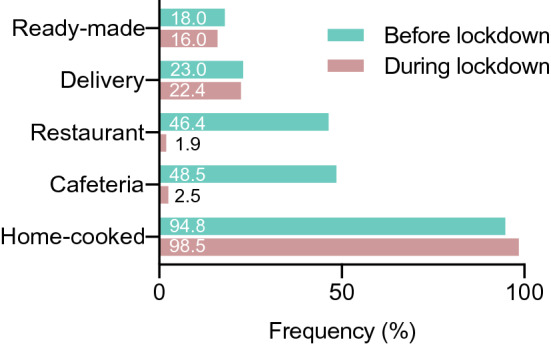
Food procurement before and during lockdown: Participants were asked in multiple choice questions to name their main ways of food procurement before and during COVID-19 lockdown. Home cooking is the main food source before (94.8%, *n* = 1861) and during (98.5%, *n* = 1935) lockdown. A dramatic decline was seen in restaurants (46.4%, *n* = 911 to 1.9%, *n* = 37) and cafeterias (48.5%, *n* = 953–2.5%, *n* = 50). No changes were seen for food deliveries or readymade food (18.0, *n* = 353–16.0, *n* = 315)

Since the implementation of lockdown, food procurement has changed dramatically among study participants (Fig. 2). While home-cooked food remains the most frequently reported source of food (94.8%, *n* = 1861 before, and 98.5%, *n* = 1935, during lockdown), the number of people visiting restaurants (46.4%, *n* = 911–1.9%, *n* = 37) or cafeterias (48.5%, *n* = 953–2.5%, *n* = 50) decreased starkly.

Almost a quarter of participants stated to be demanding food delivery services before lockdown. Although the overall fraction did not change during lockdown (23.0%, *n* = 452, vs. 22.4%, *n* = 440), 234 (51.8%) participants stopped ordering food. While obese participants (odds ratio (OR), 1.56 [95% confidence interval (CI), 1.06–2.25], *p* = 0.03) and smokers (OR, 2.43, [95% CI 1.53–3.76], *p* < 0.01) as well as participants with changed amounts of sports activities were more likely to do so, age, gender or the change in amount of food consumed during lockdown had no influence. No predictors for starting to use food delivery could be identified (Supplemental Table S1).

Readymade food was consumed by 353 participants (18.0%) before and 315 (16.0%) during lockdown. 146 (41.4%) participants stopped consuming readymade food during lockdown. Abandoning readymade food was positively influenced by an increase in sports activities (OR, 2.30 [95% CI 1.42–3.87], *p* < 0.01) and a reduced amount of food during lockdown (OR, 1.64 [95% CI 1.04–2.55], *p* = 0.03). Factors influencing the start of using readymade food included male sex (OR, 1.74 [95% CI 1.16–2.59], p < 0.01), BMI > 25 kg/m^2^ (OR, 1.99 [1.19–3.22], *p* < 0.01), decreased sports activities (OR, 2.22 [95% CI 1.34–3.88], *p* < 0.01) and increased mental stress (OR, 1.66 [1.10–2.55], *p* = 0.02, see Supplemental Table S2).

### Amount of food consumption after lockdown

**Fig. 3:**
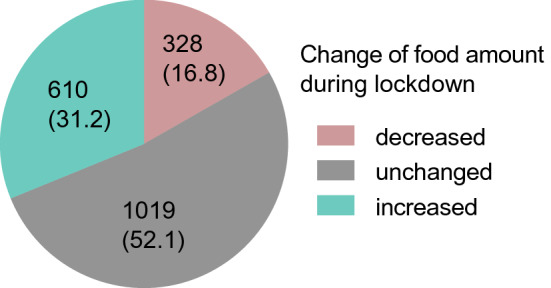
Quantification of food quantity before and during lockdown: (A) participants were asked if the amount of food intake had changed after the lockdown. Most participants, 52.1% (*n* = 1019) reported to not have altered their food amount. 16.8% (*n* = 328) stated to eat less and 31.2% (*n* = 610) to eat more compared to before the lockdown

Most participants (52.1%, *n* = 1019) stated to not have altered the amount of food they consume during lockdown (Fig. 3). Almost one-third of study subjects stated to be eating more (31.2%, *n* = 610), whereas 16.8% (*n* = 328) of the participants ate less compared to the time before lockdown. Characteristics of these three subgroups are given in Supplemental Table S3. Descriptive analyses on changes in consumption (Table 1) showed that an increase was more frequent in women (*n* = 453, 32.8% vs *n* = 147, 26.6%) and in the age group of 17–25 years (*n* = 480, 31.7%).

**Table 1:** Bivariate analyses of changes in food consumption since lockdown and potential predictor variables

Changes in food amount					
	*n*_missing_	Decreased	Unchanged	Increased	*p* value
		% (*n*)			
Gender					
Male	22	17.6 (97)	55.8 (308)	26.6 (147)	**0.031**
Female		16.5 (228)	50.8 (702)	32.8 (453)	
Age					
17–25	3	16.7 (252)	51.6 (781)	31.7 (480)	0.770
26–35		17.0 (71)	53.0 (221)	30.0 (125)	
36–50		16.7 (4)	62.5 (15)	20.8 (5)	
BMI					
BMI < 20 kg/m^2^	12	16.3 (74)	54.1 (245)	29.6 (134)	**0.018**
BMI 20–25 kg/m^2^		15.8 (195)	53.0 (655)	31.2 (386)	
BMI > 25 kg/m^2^		22.7 (58)	43.0 (110)	34.4 (88)	
Changes in sports activity since lockdown					
Decreased	14	18.2 (158)	48.8 (423)	33.0 (286)	**0.001**
Unchanged		11.4 (50)	59.5 (262)	29.1 (128)	
Increased		18.4 (117)	51.3 (326)	30.3 (193)	
Changes in alcohol consumption since lockdown					
Decreased	14	18.8 (153)	49.1 (400)	32.1 (262)	** < 0.001**
Unchanged		16.4 (140)	57.3 (489)	26.3 (225)	
Increased		12.8 (35)	44.9 (123)	42.3 (116)	
Smoking					
Yes	24	18.5 (22)	39.5 (47)	42.0 (50)	**0.012**
No		16.5 (300)	52.9 (960)	30.5 (554)	
Mental stress					
Decreased	20	17.3 (58)	55.8 (187)	26.9 (90)	**0.015**
Unchanged		16.4 (137)	54.3 (453)	29.3 (244)	
Increased		17.1 (131)	47.5 (365)	35.4 (272)	

Data are given as percentage (absolute numbers). *p* values from Chi-squared test

Bold values indicate statistically significant *p* values < 0.05

*BMI* body mass index

### Determinants of a changed food amount

**Fig. 4:**
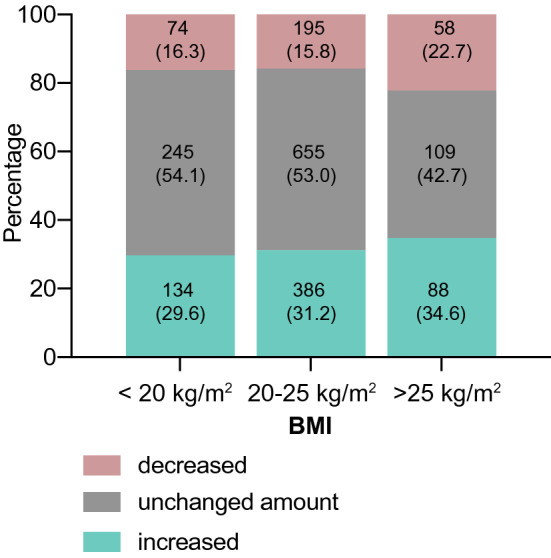
Alteration of food amount stratified by BMI: participants were stratified by BMI. Numbers are given in Table 2. Alterations in food intake were significantly different for the three categories (*p* = 0.018). More participants with a high BMI (> 25 kg/m^2^) (57.3%, *n* = 146) than with a low BMI (< 20 kg/m^2^) (45.9%, *n* = 208) or a normal BMI (20–25 kg/m^2^) (47.0%, *n* = 581) changed the amount of food they eat compared to before implementation of lockdown

We found that participants with a BMI < 20 kg/m^2^ and people with a normal BMI did not alter their consumed food amount as much as participants with a BMI > 25 kg/m^2^, (decreased: *n* = 58, 22.7%, increased: *n* = 88, 34.6%, Fig. 4). The distribution of changes in food consumption was almost similar in participants with a decreased or increased amount of sports activity. An increased alcohol consumption (42.3%), smoking (42.0%) and mental stress (35.4%) during lockdown were associated with eating more.

**Fig. 5:**
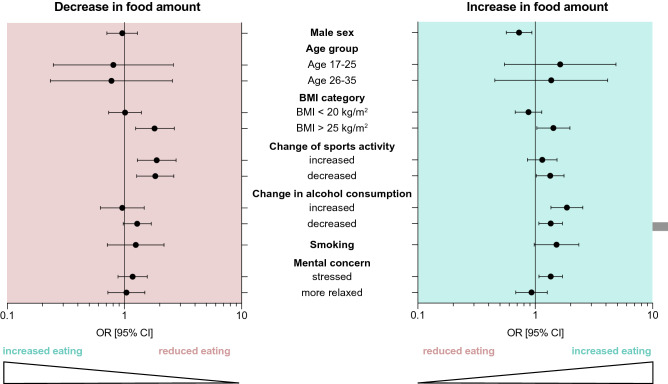
Forest plots depicting odds ratios for variables impacting a decreased or an increased food amount. Subgroups of decreased and increased overall food intake were referenced to a constant food intake. Odds ratios (OR) and 95% confidence intervals (CI) are depicted with balls and bars, respectively, for the different categories. Changes in food consumption were promoted by gender, BMI, sports activity, alcohol consumption and smoking status. Detailed data and reference categories are shown in Table 2

In a bivariate analysis (Table 1) sex, BMI, changes in sports activity and alcohol consumption as well as smoking and stress showed a significant correlation (*p*_chi2_ < 0.05) with the outcome variable (changes in food amount consumed). The regression model (Table 2, Fig. 5) confirmed the observation that a BMI > 25 kg/m^2^ had a significant effect on an altered food amount for both, decreased (odds ratio (OR), 1.8, [95% confidence interval (CI), 1.3–2.7]), and increased overall food intake (OR, 1.4 [CI 1.3–2.0]). Furthermore, a reduction of sports activity positively affected the chance of a modified food intake (decreased consumption, OR, 1.8 [CI 1.3–2.6], increased consumption, OR, 1.3 [CI 1.2–1.8]). However, the enhancement of sports only impacted a decrease in consumption (OR, 1.9 [CI 1.3–2.8]). For increased consumption, we identified male gender as a protective factor (OR, 0.7 [CI 0.6–0.9]), while changes in alcohol drinking patterns made an increased food intake significantly more likely (reduced alcohol amount, OR, 1.4 [CI 1.1–1.7], increased alcohol, OR, 1.9 [CI 1.4–2.5]).

**Table 2:** Multinomial regression model of changes in food consumption and potential predictor variables

*Changes in food consumption* *[reference: constant food consumption]*					
Decreased food consumption					
	B	OR [Exp(B)]	*p* value	Lower 95% CI	Upper 95% CI
Sex *[ref.: female]*					
Male	− 0.042	0.959	0.782	0.710	1.293
Age *[ref.: 36–50 years]*					
17–25	− 0.215	0.806	0.721	0.248	2.626
26–35	− 0.253	0.776	0.679	0.234	2.574
BMI^1^ *[ref.: BMI 20–25* kg/m^2^*]*					
BMI < 20 kg/m^2^	0.013	1.013	0.939	0.734	1.398
BMI > 25 kg/m^2^	0.594	1.811	**0.002**	1.248	2.672
Changes in sports activity since lockdown *[ref.: constant]*					
Reduced	0.607	1.835	**0.001**	1.274	2.643
Increased	0.636	1.889	**0.001**	1.293	2.762
Changes in alcohol consumption since lockdown *[ref.: constant]*					
Reduced	0.252	1.286	0.074	0.976	1.695
Increased	− 0.039	0.961	0.857	0.625	1.478
Smoking *[ref.: non-Smoker]*					
Smoker	0.223	1.250	0.431	0.717	2.179
Mental stress *[ref.: constant]*					
Reduced	0.040	1.041	0.829	0.724	1.496
Increased	0.163	1.177	0.266	0.883	1.568

Increase in food consumption					
	B	OR [Exp(B)]	*p* value	Lower 95% CI	Upper 95% CI
Sex *[ref.: female]*					
Male	− 0.137	0.728	**0.013**	0.567	0.936
Age *[ref.: 36–50 years]*					
17–25	0.490	1.632	0.380	0.547	4.867
26–35	0.313	1.368	0.579	0.452	4.138
BMI^1^ *[ref.: BMI 20–25* kg/m^2^*]*					
BMI < 20 kg/m^2^	− 0.132	0.876	0.319	0.675	1.136
BMI > 25 kg/m^2^	0.356	1.427	**0.032**	1.032	1.974
Changes in sports activity since lockdown *[ref.: constant]*					
Reduced	0.293	1.340	**0.035**	1.022	1.758
Increased	0.137	1.146	0.354	0.859	1.531
Changes in alcohol consumption since lockdown *[ref.: constant]*					
Reduced	0.0302	1.353	**0.010**	1.074	1.704
Increased	0.620	1.859	**0.000**	1.362	2.538
Smoking *[ref.: non-Smoker]*					
Smoker	0.417	1.518	0.062	0.979	2.353
Mental stress *[ref.: constant]*					
Reduced	− 0.073	0.929	0.641	0.682	1.266
Increased	0.304	1.355	**0.010**	1.076	1.706

Bold values indicate statistically significant *p* values < 0.05

*OR* odds ratio, *CI* confidence interval, *BMI* body mass index

### Change in food categories

**Fig. 6:**
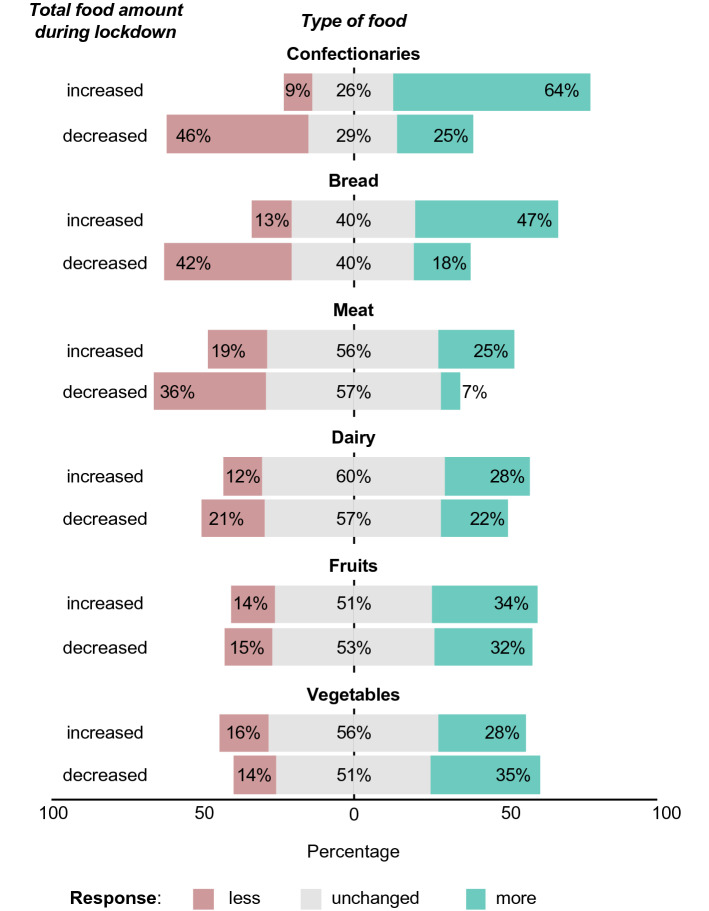
Change in food categories in participants with an increased or decreased overall food intake. In all participants, bread and confectionary intake changed more than other food categories. Among people reporting to have increased their food amount, confectionary consumption increased more compared to people who reported to have decreased their total food amount (64.4%, *n* = 389, vs. 25.0%, *n* = 82, *p* < 0.001). Significant differences were also seen for the amount of meat and bread consumed. No differences were observed for vegetables or fruits. Dairy product consumption was affected the least (eat less, 57.2%, *n* = 187, vs. eat more, 59.6%, *n* = 362, did not change the amount)

The observed changes in the amount of consumed food are mirrored in the diet of the participants (Fig. 6). The consumption of confectionary and bread increased more clearly in participants consuming an increased amount of food compared to before the lockdown. The confectionary consumption increased in 64.4% (*n* = 389) and 25.0% (*n* = 82) of participants for people with an overall increased or decreased food consumption, respectively. This was influenced by the amount of other types of food consumed. Interestingly, while an increase in most food categories was a predictor for higher confectionary consumption, this was the opposite for fruit and vegetable consumption (Supplemental Table S4).

The bread consumption was increased in 46.8% (*n* = 284) among people with increased food amounts and in only 18.6% (*n* = 61) among people eating less. Similar numbers were observed for the meat consumption (increase in 24.5% (*n* = 146) and 6.8% (*n* = 22), respectively). No relevant differences were found in fruit, vegetable or dairy product consumption. In fact, more than half the participants reported an unchanged amount in these categories. Changes in food categories among people who stated to not have changed the amount of food they consume are presented in Supplemental Fig. S1. There were hardly any differences between men and women (Supplemental Fig. S2). BMI groups had no relevant effect on the types of food consumed (data not shown).

## Discussion

The COVID-19 pandemic dramatically affects daily life for citizens all over the world. To the best of our knowledge, this is the first study investigating the immediate impact of COVID-19 pandemic lockdown on food procurement and dietary habits in a cohort of young adults. In this cohort of almost 2000 young adults, we were able to show the following. 1. Government lockdown significantly altered the amount of food intake. 16.8% of the participants reported a reduced daily food intake compared to 31.2% who showed an increase. 2. The food amount was mainly influenced by gender, BMI, sports activity, smoking, mental stress and alcohol consumption. 3. Food procurement and composition changed after implementation of the lockdown, with an enhanced consumption of confectionary.

On the 21st of March, the government in Bavaria implemented a general lockdown and activities outside the place of residence were restricted [9]. To slow the transmission of COVID-19, people were advised to do food shopping as infrequently as possible. However, since the beginning of the crisis, a lot of people reacted with “panic buying” and stocked up groceries [10].

In a first step, we asked our cohort to provide data on the change of daily consumed food amount during COVID-19 pandemic. Interestingly, about one-third of our study subjects reported to have increased the daily food amount, which correlates with the current literature [8, 11, 12]. There are several possible explanations for this response. In a multinominal regression, we identified mental stress, BMI > 25 kg/m^2^, altered sports activity and altered alcohol consumption as risk factors for increased eating.

First, changes in eating patterns are partly triggered by augmented psychological stress during the COVID-19 crisis [13]. Our results show a higher risk for an increased food intake in people who state to be mentally stressed. Similarly, Pellegrini et al. could demonstrate a weight gain during the COVID-19 lockdown of over 3 kg in patients with self-perceived anxiety during the crisis [14]. Data from the Midlife in the United States (MIDUS) study containing a representative cohort of adults revealed that psychosocial stress contributes to changes in dietary behaviors that again results in significant weight gain [15]. Interestingly, weight gain in that cohort was more pronounced in people with higher body mass indexes.

In our data, participants with BMI > 25 kg/m^2^ reported more frequently to have increased food intake compared to the subgroups of participants with a normal or reduced BMI. In the regression model, BMI > 25 kg/m^2^ was found to be an independent risk factor for an increased food intake (OR 1.8 [CI 1.3–2.7]). Similar to the former MIDUS study, individuals with a high BMI have been shown to increase their food consumption considerably and gained more weight during COVID-19 lockdown [11]. However, this contrasts with the comprehensive analysis of Di Renzo et al. who reported no difference in BMI in study participants eating more [8].

Nevertheless, a higher BMI has been found to be associated with a lower level of sports activity and sports activity is an important factor for a reduction and maintenance of body weight [16, 17]. In two cohort studies of obese patients in Italy during COVID-19 lockdown, a further decrease in sports activity was observed and resulted in a significant additional weight gain [7, 14]. In line with this, in our study, a self-reported modification of sports activity was associated with lower food intake and an increase in sports activity was found to predict a lower food intake (OR 1.9 [CI 1.3–2.8]).

These data are also important from a clinical perspective, since obesity significantly increases the risk of developing severe pneumonia in COVID-19 infections [18], and obesity is a common risk factor for respiratory failure leading to invasive mechanical ventilation [19].

Previous studies have assessed the impact of natural disasters on food and nutrition management [20]. A retrospective cohort study evaluated the impact of the devastating earthquake and tsunami in Japan in 2011 in preschool children. They found a significant increase in BMIs 1 month after the earthquake [21]. Moreover, in 200 adult subjects, results of physical examinations and laboratory tests after the Great East Japan Earthquake revealed higher values of body weight, body mass index, waist circumference and HbA1c compared with pre-earthquake levels [22].

Another important information from our subjects is the change of food procurement during the pandemic lockdown. Most subjects reported to self-prepare and cook food at home—as they did before lockdown implementation. Other studies could demonstrate a reduction of delivery food intake [8]. Interestingly, in this study, there was no relevant change in the overall frequency of people getting their food from a takeaway or from restaurants offering a delivery service, but a considerable fraction of participants changed their attitude towards readymade food and food delivery services. Male sex, high BMI and decreased sports activities during lockdown were identified as factors promoting the consumption of readymade food.

Among participants of our study reporting to consume more food after lockdown, there was a detectable shift toward carbohydrate-containing food such as confectionaries and bread. This observation has been made in Italy [12] and Poland [11], too. Unfortunately, our data also indicate that increased confectionary consumption had a higher chance of decreased fruit and vegetable consumption. An increase of carbohydrate consumption may promote cardiovascular diseases, obesity, and diabetes [23]. A previous large cohort study could further demonstrate a strong association of a high caloric diet with higher risk of all-cause mortality [24]. However, long-term effects of the observed nutrition shift on morbidity and mortality are not assessable at this time.

Our study has some limitations. It is a cross-sectional study and the resulting changes in weight/BMI in this study cohort cannot be reassessed in the future. Conclusions drawn are based on self-reported amounts of food consumption. Therefore, the analyses included in the study need to be interpreted with caution. The analysis focused on young adults who are registered at Bavarian Universities and does not fully represent young adults throughout Germany. However, the homogenous study cohort provides valuable insights into COVID-19 pandemic related changes of food and nutrition management in healthy young individuals in Germany.

In summary, we were able to show for the first time a change in the food amount, its underlying determinants, acquisition of food as well as food composition among healthy young adults during the COVID-19 crisis. Further longitudinal studies investigating long-term effects are warranted.

## Electronic supplementary material

Below is the link to the electronic supplementary material.

(DOCX 48 kb)

(DOCX 21 kb)

Fig. S1 Change in food categories in participants with an unchanged overall food intake. Among participants with unchanged food amounts during lockdown, confectionary consumption has changed in 50% (increased consumption, n = 291, 27.2%, unchanged consumption, n = 495, 50.5%, decreased consumption, n = 212, 22.3%, missing, n = 11). The other categories (bread, meat, dairy, fruits, vegetables) were affected less. If participants stated to have changed their habits here, meat consumption was mostly reduced while increased intakes were observed for dairy, fruits and vegetables. (PS 778 kb)

Fig. S2 Type of food among male and female participants with an increased or decreased overall food intake. Hardly any differences could be found between male and female participants who stated to have altered their overall food intake regarding changed amounts of different types of food. More male than female participants changed the amount of dairy products (50% vs. 38%) and usually increased their consumption here (PS 746 kb)
