# Supplementary material for: Altered nutrition behavior during COVID-19 pandemic lockdown in young adults
Source: Eur J Nutr. 2020 Dec 1;60(5):2593–602. doi: 10.1007/s00394-020-02435-6 (PMC7705857; doi:10.1007/s00394-020-02435-6)
Supplement: Supplementary file 1 — (DOCX 48 kb) [file 394_2020_2435_MOESM1_ESM.docx]

**Supplementary Table S1:** Univariate analyses to identify predictors for beginning or stopping using food delivery services

|  | **Stop using food delivery** | | | | | **Start using food delivery** | | | | |  |
| --- | --- | --- | --- | --- | --- | --- | --- | --- | --- | --- | --- |
|  | FALSE | TRUE | OR [95% CI] | p.ratio | p.overall | FALSE | TRUE | OR [95% CI] | p.ratio | p.overall | N |
|  | N=1730 | N=234 |  |  |  | N=1742 | N=222 |  |  |  |  |
| **sex:** |  |  |  |  | 0.240 |  |  |  |  | 0.999 | 1941 |
| **M** | 480 (28.1%) | 74 (32.0%) | 1.21 [0.90-1.62] | 0.213 |  | 492 (28.6%) | 62 (28.3%) | 0.99 [0.72-1.34] | 0.943 |  |  |
| **F** | 1230 (71.9%) | 157 (68.0%) | Ref. | Ref. |  | 1230 (71.4%) | 157 (71.7%) | Ref. | Ref. |  |  |
| **Age** | 23.3 (4.10) | 23.3 (3.58) | 1.00 [0.97-1.03] | 0.994 | 0.994 | 23.3 (4.03) | 23.5 (4.11) | 1.01 [0.98-1.05] | 0.427 | 0.435 | 1960 |
| **Age group:** |  |  |  |  | 0.366 |  |  |  |  | 0.965 | 1959 |
| **17-25 years** | 1341 (77.7%) | 177 (75.6%) | 0.85 [0.62-1.18] | 0.327 |  | 1347 (77.5%) | 171 (77.4%) | 0.97 [0.70-1.38] | 0.879 |  |  |
| **26-35 years** | 361 (20.9%) | 56 (23.9%) | Ref. | Ref. |  | 369 (21.2%) | 48 (21.7%) | Ref. | Ref. |  |  |
| **36-50 years** | 23 (1.33%) | 1 (0.43%) | 0.32 [0.01-1.55] | 0.190 |  | 22 (1.27%) | 2 (0.90%) | 0.75 [0.11-2.66] | 0.692 |  |  |
| **BMI group:** |  |  |  |  | 0.062 |  |  |  |  | 0.088 | 1947 |
| **20-25 kg/m^2^** | 1099 (64.1%) | 138 (59.5%) | Ref. | Ref. |  | 1084 (62.7%) | 153 (69.9%) | Ref. | Ref. |  |  |
| **>25 kg/m^2^** | 215 (12.5%) | 42 (18.1%) | 1.56 [1.06-2.25] | 0.025 |  | 230 (13.3%) | 27 (12.3%) | 0.84 [0.53-1.27] | 0.409 |  |  |
| **<20 kg/m^2^** | 401 (23.4%) | 52 (22.4%) | 1.03 [0.73-1.44] | 0.845 |  | 414 (24.0%) | 39 (17.8%) | 0.67 [0.46-0.96] | 0.028 |  |  |
| **Mental stress** |  |  |  |  | 0.274 |  |  |  |  | 0.280 | 1943 |
| **unchanged** | 741 (43.3%) | 96 (41.2%) | Ref. | Ref. |  | 749 (43.5%) | 88 (40.0%) | Ref. | Ref. |  |  |
| **decreased** | 287 (16.8%) | 49 (21.0%) | 1.32 [0.90-1.90] | 0.148 |  | 302 (17.5%) | 34 (15.5%) | 0.96 [0.62-1.45] | 0.851 |  |  |
| **increased** | 682 (39.9%) | 88 (37.8%) | 1.00 [0.73-1.35] | 0.980 |  | 672 (39.0%) | 98 (44.5%) | 1.24 [0.91-1.69] | 0.167 |  |  |
| **Smoker** | 92 (5.39%) | 28 (12.1%) | 2.43 [1.53-3.76] | <0.001 | <0.001 | 112 (6.52%) | 8 (3.64%) | 0.55 [0.24-1.08] | 0.085 | 0.128 | 1939 |
| **Sports activity** |  |  |  |  | 0.022 |  |  |  |  | 0.937 | 1949 |
| **unchanged** | 407 (23.7%) | 36 (15.6%) | Ref. | Ref. |  | 391 (22.6%) | 52 (23.6%) | Ref. | Ref. |  |  |
| **less** | 754 (43.9%) | 113 (48.9%) | 1.69 [1.15-2.54] | 0.007 |  | 771 (44.6%) | 96 (43.6%) | 0.94 [0.66-1.35] | 0.715 |  |  |
| **more** | 557 (32.4%) | 82 (35.5%) | 1.66 [1.11-2.53] | 0.014 |  | 567 (32.8%) | 72 (32.7%) | 0.95 [0.65-1.40] | 0.809 |  |  |
| **Food amount  during lockdown** |  |  |  |  | 0.900 |  |  |  |  | 0.808 | 1957 |
| **less** | 287 (16.6%) | 41 (17.7%) | Ref. | Ref. |  | 288 (16.6%) | 40 (18.2%) | Ref. | Ref. |  |  |
| **unchanged** | 901 (52.2%) | 118 (50.9%) | 0.91 [0.63-1.35] | 0.648 |  | 908 (52.3%) | 111 (50.5%) | 0.88 [0.60-1.30] | 0.513 |  |  |
| **more** | 537 (31.1%) | 73 (31.5%) | 0.95 [0.63-1.44] | 0.807 |  | 541 (31.1%) | 69 (31.4%) | 0.92 [0.61-1.40] | 0.684 |  |  |

OR, Odds Ratio; CI, Confidence Interval; BMI, Body Mass Index; Ref., reference category; N, number analyzed.

**Supplementary Table S2:** Univariate analyses to identify predictors for beginning or stopping consuming readymade food.

|  | **Stop consuming readymade food** | | | | | **Start consuming readymade food** | | | | |  |
| --- | --- | --- | --- | --- | --- | --- | --- | --- | --- | --- | --- |
|  | FALSE | TRUE | OR [95% CI] | p.ratio | p.overall | FALSE | TRUE | OR [95% CI] | p.ratio | p.overall | N |
|  | N=1818 | N=146 |  |  |  | N=1856 | N=108 |  |  |  |  |
| **sex:** |  |  |  |  | 0.328 |  |  |  |  | 0.008 | 1941 |
| **M** | 507 (28.2%) | 47 (32.4%) | 1.22 [0.84-1.75] | 0.286 |  | 511 (27.9%) | 43 (40.2%) | 1.74 [1.16-2.59] | 0.008 |  |  |
| **F** | 1289 (71.8%) | 98 (67.6%) | Ref. | Ref. |  | 1323 (72.1%) | 64 (59.8%) | Ref. | Ref. |  |  |
| **Age** | 23.4 (4.07) | 22.9 (3.60) | 0.97 [0.92-1.01] | 0.159 | 0.119 | 23.3 (4.01) | 23.8 (4.53) | 1.03 [0.98-1.08] | 0.204 | 0.257 | 1960 |
| **Age group:** |  |  |  |  | 0.949 |  |  |  |  | 0.359 | 1959 |
| **17-25 years** | 1406 (77.5%) | 112 (77.2%) | 0.96 [0.64-1.46] | 0.827 |  | 1439 (77.7%) | 79 (73.1%) | 0.79 [0.51-1.26] | 0.316 |  |  |
| **26-35 years** | 385 (21.2%) | 32 (22.1%) | Ref. | Ref. |  | 390 (21.1%) | 27 (25.0%) | Ref. | Ref. |  |  |
| **36-50 years** | 23 (1.27%) | 1 (0.69%) | 0.59 [0.02-2.96] | 0.595 |  | 22 (1.19%) | 2 (1.85%) | 1.40 [0.20-5.15] | 0.683 |  |  |
| **BMI group:** |  |  |  |  | 0.471 |  |  |  |  | 0.015 | 1947 |
| **20-25 kg/m^2^** | 1151 (63.9%) | 86 (58.9%) | Ref. | Ref. |  | 1176 (63.9%) | 61 (57.0%) | Ref. | Ref. |  |  |
| **>25 kg/m^2^** | 236 (13.1%) | 21 (14.4%) | 1.20 [0.71-1.93] | 0.486 |  | 233 (12.7%) | 24 (22.4%) | 1.99 [1.19-3.22] | 0.009 |  |  |
| **<20 kg/m^2^** | 414 (23.0%) | 39 (26.7%) | 1.26 [0.84-1.86] | 0.253 |  | 431 (23.4%) | 22 (20.6%) | 0.99 [0.59-1.61] | 0.964 |  |  |
| **Mental stress** |  |  |  |  | 0.832 |  |  |  |  | 0.006 | 1943 |
| **unchanged** | 778 (43.2%) | 59 (41.0%) | Ref. | Ref. |  | 798 (43.5%) | 39 (36.1%) | Ref. | Ref. |  |  |
| **decreased** | 309 (17.2%) | 27 (18.8%) | 1.16 [0.71-1.84] | 0.554 |  | 325 (17.7%) | 11 (10.2%) | 0.70 [0.34-1.34] | 0.293 |  |  |
| **increased** | 712 (39.6%) | 58 (40.3%) | 1.07 [0.74-1.57] | 0.710 |  | 712 (38.8%) | 58 (53.7%) | 1.66 [1.10-2.55] | 0.016 |  |  |
| **Smoker** | 108 (6.02%) | 12 (8.33%) | 1.44 [0.73-2.58] | 0.276 | 0.352 | 109 (5.95%) | 11 (10.2%) | 1.81 [0.89-3.35] | 0.097 | 0.117 | 1939 |
| **Sports activity** |  |  |  |  | <0.001 |  |  |  |  | <0.001 | 1949 |
| **unchanged** | 421 (23.3%) | 22 (15.3%) | Ref. | Ref. |  | 425 (23.1%) | 18 (16.7%) | Ref. | Ref. |  |  |
| **less** | 814 (45.1%) | 53 (36.8%) | 1.24 [0.75-2.11] | 0.404 |  | 792 (43.0%) | 75 (69.4%) | 2.22 [1.34-3.88] | 0.002 |  |  |
| **more** | 570 (31.6%) | 69 (47.9%) | 2.30 [1.42-3.87] | 0.001 |  | 624 (33.9%) | 15 (13.9%) | 0.57 [0.28-1.15] | 0.114 |  |  |
| **Food amount  during lockdown** |  |  |  |  | 0.060 |  |  |  |  | 0.177 | 1957 |
| **unchanged** | 956 (52.8%) | 63 (43.2%) | Ref. | Ref. |  | 971 (52.5%) | 48 (44.4%) | Ref. | Ref. |  |  |
| **less** | 296 (16.3%) | 32 (21.9%) | 1.64 [1.04-2.55] | 0.033 |  | 310 (16.8%) | 18 (16.7%) | 1.18 [0.66;2.03] | 0.565 |  |  |
| **more** | 559 (30.9%) | 51 (34.9%) | 1.38 [0.94-2.03] | 0.099 |  | 568 (30.7%) | 42 (38.9%) | 1.50 [0.97;2.29] | 0.067 |  |  |

OR, Odds Ratio; CI, Confidence Interval; BMI, Body Mass Index; Ref., reference category; N, number analyzed.

**Supplementary Table S3:** Study demographics, stratified for change in food amount: All numbers are absolute number and percentage or mean ± standard deviation.

|  | Total (n=1957) | Change in food amount | | |  | p value |
| --- | --- | --- | --- | --- | --- | --- |
|  |  | **decreased (n=328)** | **unchanged (n=1019)** | **increased (n=610)** | **n_missing_** |  |
| Female gender | 1383 (71.5%) | 228 (70.2%) | 702 (69.5%) | 453 (75.5%) | 22 | 0.03 |
| Age (years) | 23.4 ±4.0 | 23.5 +4.3 | 23.4 ±4.0 | 23.2 ±3.9 | 2 | 0.38 |
| BMI (kg/m^2^) | 22.1 ±4.5 | 22.5 ±3.3 | 21.9 ±2.8 | 22.4 ±6.7 | 15 | 0.02 |

Chi-squared test was used for comparison of categorial data, Wilcox test was used for comparison of metric data. Data on change in food amount were missing for 7 participants.

**Supplementary Table S4:** Univariate analysis of factors predicting an increased confectionary consumption during lockdown.

|  | Less or  unchanged amount  of confectionary | More confectionary | OR [95% CI] | p value | N |
| --- | --- | --- | --- | --- | --- |
|  | N=1174 | N=766 |  |  |  |
| **Vegetables** |  |  |  |  | 1940 |
| less | 91 (7.75%) | 131 (17.1%) | 2.06 [1.54-2.76] | <0.001 |  |
| unchanged | 701 (59.7%) | 490 (64.0%) | Ref. | Ref. |  |
| more | 382 (32.5%) | 145 (18.9%) | 0.54 [0.43-0.68] | <0.001 |  |
| **Fruits** |  |  |  |  | 1938 |
| less | 101 (8.62%) | 128 (16.7%) | 1.98 [1.49-2.65] | <0.001 |  |
| unchanged | 691 (59.0%) | 441 (57.6%) | Ref. | Ref. |  |
| more | 380 (32.4%) | 197 (25.7%) | 0.81 [0.66-1.00] | 0.051 |  |
| **Dairy** |  |  |  |  | 1932 |
| less | 154 (13.2%) | 74 (9.71%) | 0.76 [0.56-1.03] | 0.073 |  |
| unchanged | 796 (68.0%) | 502 (65.9%) | Ref. | Ref. |  |
| more | 220 (18.8%) | 186 (24.4%) | 1.34 [1.07-1.68] | 0.011 |  |
| **Meat** |  |  |  |  | 1906 |
| less | 299 (25.8%) | 133 (17.8%) | 0.69 [0.55-0.87] | 0.002 |  |
| unchanged | 732 (63.3%) | 471 (62.9%) | Ref. | Ref. |  |
| more | 126 (10.9%) | 145 (19.4%) | 1.79 [1.37-2.33] | <0.001 |  |
| **Bread** |  |  |  |  | 1935 |
| less | 340 (29.1%) | 91 (11.9%) | 0.42 [0.32-0.55] | <0.001 |  |
| unchanged | 608 (52.0%) | 388 (50.7%) | Ref. | Ref. |  |
| more | 221 (18.9%) | 287 (37.5%) | 2.03 [1.64-2.53] | <0.001 |  |

OR, Odds Ratio; CI, Confidence Interval; BMI, Body Mass Index; Ref., reference category; N, number analyzed.
