# Supplementary material for: Altered nutrition behavior during COVID-19 pandemic lockdown in young adults
Source: Eur J Nutr. 2020 Dec 1;60(5):2593–602. doi: 10.1007/s00394-020-02435-6 (PMC7705857; doi:10.1007/s00394-020-02435-6)
Supplement: Supplementary file 2 — (DOCX 21 kb) [file 394_2020_2435_MOESM2_ESM.docx]

**Altered Nutrition Behavior
During COVID-19 Pandemic Lockdown in Young Adults**

Bruno C. Huber, MD; Julius Steffen, MD; Jenny Schlichtiger, MSc; Stefan Brunner, MD

**Appendix**

**Online Questionnaire (translation)**

| 1. **Gender** | male  female |
| --- | --- |
| 1. **Age** | number |
| 1. **Height** | number in cm |
| 1. **Weight** | number in kg |
| 1. **I’m mentally stressed because of the Coronavirus pandemic** | less  unchanged  more |
| 1. **How much sports do you do, compared to before lockdown?** | less  unchanged  more |
| 1. **How has your diet changed since implementation of lockdown?** |  |
| - - **Overall food amount** | less  unchanged  more |
| - - **Vegetables** | less  unchanged  more |
| - - **Fruit** | less  unchanged  more |
| - - **Meat** | less  unchanged  more |
| - - **Dairy products** | less  unchanged  more |
| - - **Bread** | less  unchanged  more |
| - - **Confectionary** | less  unchanged  more |
| - - **Alcohol** | less  unchanged  more |
| 1. **Food procurement BEFORE lockdown** | (multiple choice) |
| - - Ready made dishes |  |
| - - Home-cooked |  |
| - - Restaurants/cafes |  |
| - - Delivery/take away |  |
| - - Cafeteria/mensa |  |
| 1. **Food procurement DURING lockdown** | (multiple choice) |
| - - Ready made dishes |  |
| - - Home-cooked |  |
| - - Restaurants/cafes |  |
| - - Delivery/take away |  |
| - - Cafeteria/mensa |  |
| 1. **Do you smoke?** | yes  no |
| - - **If yes, do you smoke** … **since implementation of exit restrictions?** | …less  …unchanged  …more |
